# T3 Pharmacogenetic Gene-drug Associations in Pediatric Burn and Surgery Patients

**DOI:** 10.1093/jbcr/irac012.002

**Published:** 2022-03-23

**Authors:** Kristin N Grimsrud, Ryan Davis, Clifford Tepper, Tina L Palmieri

**Affiliations:** University of California, Davis, Davis, California; University of California, Davis, Sacramento, California; University of California, Davis, Sacramento, California; UC Davis and Shriners Hospitals for Children Northern California, Sacramento, California

## Abstract

**Introduction:**

Management of critically ill patients requires simultaneous administration of many medications. These patients may have co-morbidities and drug-drug interactions which may result in decreased drug efficacy or increased risk of adverse reactions. Children are of particular concern as they may be even more sensitive to adverse drug reactions. Current practices rely on a one-size-fits-all approach for most medication dosing. Alternatively, precision medicine evaluates an individual’s genetic profile and adjusts individual therapeutic dosing. Pharmacogenetic testing is not generally implemented but is reserved for addressing an established problem rather than being used proactively to optimize clinical care. A greater understanding of the frequency of clinically significant genetic variants in drug pathways and identification of the drugs most commonly impacted by genetic makeup is needed to incorporate pharmacogenetics into medical care. We hypothesize several patients will have one or more genetic variants in drug metabolizing pathways used by one or more medications administered during hospitalization. The aims of this study are to determine the frequency of abnormal genetic variants in the primary drug pathways and assess what medications may be impacted.

**Methods:**

Genetic data from 30 pediatric burn and surgery patients was collected to identify genetic variants in drug metabolizing pathways. We also evaluated drugs potentially impacted by these variants that were administered during the hospitalization. 19 whole exome and 11 whole genome sequencing datasets were analyzed using Aldy allelic decomposition software to identify haplotypes in cytochrome P450 (CYP) metabolizing enzyme genes.

**Results:**

17 patients were identified with predicted altered function in 1 or more of the following CYP genes: *2C9*, *2C19, 2D6, 3A4 and 3A5.* The majority had decreased function, except for several patients with *CYP2C19* variants with rapid or ultrarapid phenotypes. Drugs administered during hospitalization that rely on these pathways for primary metabolism include hydrocodone, oxycodone, methadone, ibuprofen, ketorolac, celebrex, diazepam, famotidine, diphenhydramine and glycopyrrolate.

**Conclusions:**

This research demonstrates that approximately 1/4 to 1/3 of patients have functionally impactful haplotypes in the primary drug metabolizing pathways. There is great need for future clinical research to further evaluate the clinical relevance of these haplotypes and the additional impact of drug-drug interactions and other clinical confounders that additionally impact drug efficacy.

